# Sensorless contact force estimation and robust impedance control for a quadrotor manipulation system

**DOI:** 10.1038/s41598-024-79606-6

**Published:** 2024-11-20

**Authors:** Alaa Khalifa, Mohamed Fanni, Ahmed Khalifa

**Affiliations:** 1 Department of Industrial Electronics and Control Engineering, Faculty of Electronic Engineering, Menoufia University, Menouf 32952, Egypt; 2 Department of Mechatronics and Robotics Engineering, Egypt-Japan University of Science and Technology, Alexandria, Egypt; 3 Cardiff School of technologies, Cardiff Metropolitan University, Cardiff, UK

**Keywords:** Aerospace engineering, Electrical and electronic engineering

## Abstract

The research on aerial manipulation systems has been increased rapidly in recent years. These systems are very attractive for a wide range of applications due to their unique features. However, dynamics, control and manipulation tasks of such systems are quite challenging because they are naturally unstable, have very fast dynamics, have strong nonlinearities, are very susceptible to parameters variations due to carrying a payload besides the external disturbances, and have complex inverse kinematics. In addition, the manipulation tasks require estimating (applying) a certain force of (at) the end-effector as well as the accurate positioning of it. Thus, in this article, a robust force estimation and impedance control scheme is proposed to address these issues. The robustness is achieved based on the Disturbance Observer (DOb) technique. Then, a tracking and performance low computational linear controller is used. For teleoperation purpose, the contact force needs to be identified. However, the current developed techniques for force estimation have limitations because they are based on ignoring some dynamics and/or requiring of an indicator of the environment contact. Unlike these techniques, we propose a technique based on linearization capabilities of DOb and a Fast Tracking Recursive Least Squares (FTRLS) algorithm. The complex inverse kinematics problem of such a system is solved by a Jacobin based algorithm. The stability analysis of the proposed scheme is presented. The algorithm is tested to achieve tracking of task space reference trajectories besides the impedance control. The efficiency of the proposed technique is enlightened via numerical simulation.

## Introduction

Recently, Unmanned Aerial Vehicles (UAVs) especially multi-rotors type, receive great attention due to their higher degree of mobility, speed and capability to access to regions that are inaccessible to ground vehicles^1,2^. However, UAV as a standalone vehicle has a limited functionality to the search and surveillance applications.

Due to their superior mobility, much interest is given to utilize them for aerial manipulation and thus the application of UAV manipulation systems have been expanded dramatically^3,4^. Applications of such systems include inspection, maintenance, power transmission lines upkeep^5^, structure assembly, firefighting, rescue operation, surveillance, or transportation in locations that are inaccessible, very dangerous or costly to be accessed from the ground^6^. Recent research has presented innovative methods for positioning, obstacle avoidance, and coordination in multi-UAV systems. A key focus is on how UAVs can achieve accurate localization without relying on GPS^7^and how they can coordinate to prevent collisions^8^, which remains a significant area of study.

Research on quadrotor-based aerial manipulation can be divided into different approaches based on the tool attached to the UAV including gripper based^9^, cables based^10,11^, multi-DoF robotic manipulator based^12,13^, multi-DoF dual-arms manipulator based^14,15^, compliant manipulator -based^16^, Hybrid rigid/elastic-joint manipulator^17^.

In the gripper/ tool-based approach, the attitude of the payload/tool is restricted to that of the quadrotor, and hence, the resulting aerial system has independent 4 DOFs; three translational DOFs and one rotational DOF (Yaw), i.e., the gripper/tool cannot posses pitch or roll rotation without moving horizontally. The second approach is to suspend a payload with cables but this approach has a drawback that the movement of the payload cannot be always regulated directly. To cope up with these limitations, another approach is developed in which a quadrotor is equipped with a robotic manipulator that can actively interact with the environment. Very few reports exist in the literature that investigate the combination of aerial vehicle with robotic manipulator. Kinematic and dynamic models of the quadrotor combined with arbitrary multi-DOF robot arm are derived using the Euler-Lagrangian formalism in^18^. In^19^, a quadrotor with light-weight manipulators, three 2-DOF arms, are tested. In^20^, an aerial manipulation using a quadrotor with a 2-DOF robotic arm is presented but with certain topology that disable the system from making arbitrary position and orientation of the end-effector. In this system, the axes of the manipulator joints are parallel to each other and parallel to one in-plane axis of the quadrotor. Thus, the system cannot achieve orientation around the second in-plane axis of the quadrotor without moving horizontally.

From the above discussion, the current introduced systems in the literature that use a gripper suffers from the limited allowable DOFs of the end-effector. The other systems have a manipulator with either two DOFs but in certain topology that disables the end-effector to track arbitrary 6-DOF trajectory, or more than two DOFs which decreases greatly the possible payload carried by the system.

In^13,21,22^, the authors propose a new aerial manipulation system that consists of 2-link manipulator, with two revolute joints whose axes are perpendicular to each other and the axis of the first joint is parallel to one in-plane axis of the quadrotor. Thus, the end-effector is able to reach arbitrary position and orientation without moving horizontally with minimum possible actuators.

In order to achieve position holding during manipulation, uncertainties and disturbances in the system such as wind, contact forces, measurement noise have to be compensated by using a robust control scheme. Disturbance Observer (DOb)-based controller is used to achieve a robust motion control^23–25^. The DOb estimates the nonlinear terms and uncertainties then compensates them such that the robotic system acts like a multi-SISO linear systems. Therefore, it is possible to rely on a standard linear controller to design the controller of the outer loop such that the system performance can be adjusted to achieve desired tracking accuracy and speed. In^26–28^, DOb-based motion control technique is applied to robotic-based systems and gives efficient results.

In the motion control of the aerial manipulator, achievement of the compliance control is very important because the compliance motion makes possible to perform flexible motion of the manipulator according to desired impedance^29^. This is very critical demand in applications such as demining and maintenance. In the compliance control, end-effector position and generated force of the manipulator are controlled according to the reaction force detected by the force sensor. In this method, the desired impedance is selected arbitrary in the controller. However, the force sensor is essential to detect the reaction force as presented in^30–32^. On line identified environment impedance has also been used for transparency in teleoperation systems^33^. These problems are more severe when environment displays sudden changes in its dynamic parameters which cannot be tracked by the identification process. In^34^, it is found that in order to faithfully convey to the operator the sense of high frequency chattering of contact between the slave and hard objects, faster identification and structurally modified methods were required. However, these methods need the measurement of force.

A control framework was introduced in^35^, characterized by its flexibility and adaptability. It employs a centralized hardware structure, while the software architecture adjusts according to the specific task. For tasks requiring precise, force-independent movements, it functions in a distributed manner. Conversely, for tasks that need compliance and force control, it operates in a decentralized manner. A new adaptive $H_{\infty }$control method for adaptive cruise control (ACC) systems has been developed using data-driven learning^36^. This approach begins by creating a continuous-time ACC system with unknown dynamics. An adaptive estimator is then designed to reconstruct these unknown dynamics using observable input/output data. An adaptive law ensures the estimated parameters convergence. A study explores the U-Model^37^, a model-independent design approach, using a quadrotor UAV as an example. The control system, based on the U-Model Dynamic Inverse control method, comprises two components: a general controller designed independently to meet predefined performance criteria, and this controller is then combined with the inverse of the model.

Several techniques are proposed to estimate the contact force and the environment dynamics. In^38^, the DOb and Recursive Least Squares (RLS) are used to estimate the environment dynamics. However, in this method, two DObs are used besides the RLS, and the estimation of contact force is activated only during the instance of contacting, thus there is a need to detect the instant at which the contact occurs. However, this is not practical approach especially if we target autonomous system. In^39–44^, several techniques are proposed to achieve force control without measuring the force. However, these techniques are based on ignoring some dynamics and external disturbances which will produce inaccurate force estimation. In^45,46^, an impedance control is designed for aerial manipulator without the need to measure/estimate the contact force. However, in such work, the authors neglect some dynamics as well as external disturbances, in addition to, the proposed algorithm is model-based and it does not have a robustness capability. In^47^, a scheme is proposed which allows a quadrotor to perform tracking tasks without a precise knowledge of its dynamics and under the effect of external disturbances and unmodeled aerodynamics. In addition, this scheme can estimate the external generalized forces. However, as the authors claim, this estimator can work perfectly with constant external disturbances. In addition, the estimated forces contain many different types of forces such as wind, payload, environment impacts, and unmodeled dynamics. Thus, it can not isolate the end-effector force only from the others. The authors in^48^ present a model-based method to estimate the external wrench of a flying robot. However, this method assumes that there are no modeling errors and no external disturbance. Moreover, it estimates the external force as one unite and it can not distinguish between external disturbance and the end-effector force which we need to calculate for teleoperation purposes. In addition, it uses a model based control which needs a full knowledge of the model.

In this article, a new scheme is proposed to cope up with these limitations of the currently developed techniques to solve the issues of this complicated multibody robotic system. Firstly, a DOb inner loop is used to estimate both the system nonlinearities and all external forces to compensate for them, as a result, the system acts like a linear decoupled MIMO system. Secondly, a fast tracking RLS algorithm is utilized with the linearization capabilities of DOb to estimate the contact force, in addition to, it enables the user to sense the contact force at the end-effector that it is not available in the current developed schemes. Thirdly, a model-free robust impedance control of the quadrotor manipulation system is implemented. The DOb is designed in the quadrotor/joint space while the impedance control is designed in the task space such that the end-effector can track the desired task space trajectories besides applying a specified environment impedance. Thus, Fourthly, a Jacobian based algorithm is proposed to transform the control signal from the task space to the quadrotor/joint space coordinates. The rigorous stability analysis of the proposed scheme is presented. Finally, the system model is simulated in MATLAB taking in to considerations all the non-idealities and based on real parameters to emulate a real system.

## System modeling

**Fig. 1:**
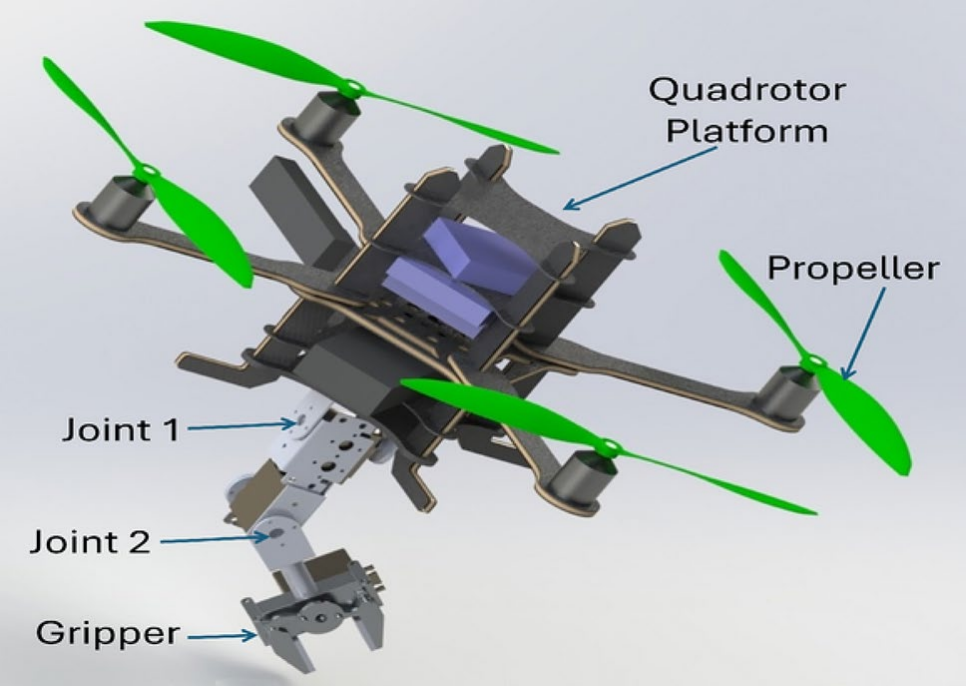
3D CAD model of the proposed quadrotor manipulation system.

Fig. 1 presents a 3D CAD model of the proposed quadrotor-based aerial manipulator. The system is composed of a manipulator mounted on the bottom center of a quadrotor.

**Fig. 2:**
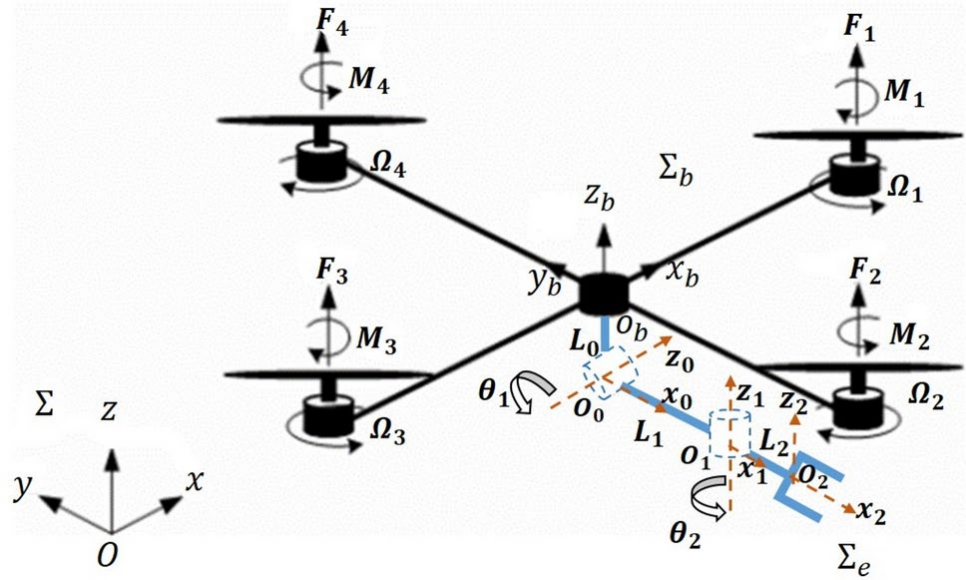
Schematic of the proposed quadrotor manipulation system with relevant frames.

System geometrical frames, which are assumed to satisfy the Denavit-Hartenberg (DH) convention, are illustrated in Fig. 2. The manipulator has two revolute joints. The axis of the first revolute joint, $z_0$, is parallel to the quadrotor *x*-axis. The axis of the second joint, $z_1$, is normal to that of the first joint and hence it is parallel to the quadrotor *y*-axis at the extended configuration. Therefore, the pitching and rolling rotation of the end-effector is allowable independently from the horizontal motion of the quadrotor. Hence, with this proposed aerial manipulator, it is possible to manipulate objects with arbitrary location and orientation. Consequently, the end-effector can make motion in 6-DOF with minimum possible number of actuators/links that is critical factor in flight.

The quadrotor components are designed to achieve a payload capacity of 500 g. Asctec pelican quadrotor is utilized as a quadrotor platform. The maximum thrust force for each rotor is 6N. The arm is designed so that the total weight of the arm is 200 g, it has a maximum reach in the range of 22, and it can carry a payload of 200 g. It has three DC motors, (HS-422 (Max torque = 0.4 N.m) for gripper, HS-5485HB (Max torque = 0.7 N.m) for joint 1, and HS-422 (Max torque = 0.4 N.m) for joint 2).

The angular velocity of each rotor *j* is $\Omega _j$ and it generates thrust force $F_j$ and drag moment $M_j$ that are given by

1 $$\begin{aligned} F_j = K_{f_j} \Omega _j^2, \end{aligned}$$

2 $$\begin{aligned} M_j = K_{m_j} \Omega _j^2, \end{aligned}$$

where $K_{f_j}$ and $K_{m_j}$ are the thrust and drag coefficients.

### Kinematics

Let $\Sigma _b$, $O_{b}$- $x_b$ $y_b$ $z_b$, represents the quadrotor body-fixed reference frame with origin at the quadrotor center of mass, see Fig. 2. Its position with respect to the world-fixed inertial reference frame, $\Sigma$, *O*- *x* *y* *z*, is given by the $(3 \times 1)$ vector $p_b=[x, y, z]^T$, while its orientation is given by the rotation matrix $R_b$ which is given by

3 $$\begin{aligned} R_b= \begin{bmatrix} C_{\psi } C_{\theta } ~ ~ & ~ ~ S_{\phi } S_{\theta } C_{\psi }-S_{\psi } C_{\phi } ~ ~ & ~ ~ S_{\psi } S_{\phi }+C_{\psi } S_{\theta } C_{\phi } \\ S_{\psi } C_{\theta } ~ ~ & ~ ~ C_{\psi } C_{\phi }+ S_{\psi } S_{\theta } S_{\phi } ~ ~ & ~ ~ S_{\psi } S_{\theta } C_{\phi }-C_{\psi } S_{\phi } \\ -S_{\theta } ~ ~ & ~ ~ C_{\theta } S_{\phi } ~ ~ & ~ ~ C_{\theta } C_{\phi } \\ \end{bmatrix}, \end{aligned}$$

where $\Phi _b$=$[\psi ,\theta ,\phi ]^T$ is the triple *ZYX* yaw-pitch-roll angles. Note that *C*(.) and *S*(.) are short notations for *cos*(.) and *sin*(.). Let us consider the frame $\Sigma _e$, $O_{2}$- $x_2$ $y_2$ $z_2$, attached to the end-effector of the manipulator, see Fig. 2. Thus, the position of $\Sigma _e$ with respect to $\Sigma$ is given by

4 $$\begin{aligned} p_e = p_b + R_b p^b_{eb}, \end{aligned}$$

where the vector $p^b_{eb}$ describes the position of $\Sigma _e$ with respect to $\Sigma _b$ expressed in $\Sigma _b$. The orientation of $\Sigma _e$ can be defined by the rotation matrix

5 $$\begin{aligned} R_e = R_b R^b_e, \end{aligned}$$

where $R^b_e$ describes the orientation of $\Sigma _e$ w.r.t $\Sigma _b$. The linear velocity ${\dot{p}}_e$ of $\Sigma _e$ in the world-fixed frame is obtained by the differentiation of (4) as

6 $$\begin{aligned} {\dot{p}}_e = {\dot{p}}_b - Skew(R_b p^b_{eb}) \omega _b + R_b {\dot{p}}^b_{eb}, \end{aligned}$$

where *Skew*(.) is the $(3 \times 3)$skew-symmetric matrix operator^49^, while $\omega _b$ is the angular velocity of the quadrotor expressed in $\Sigma$. The angular velocity $\omega _e$ of $\Sigma _e$ is expressed as

7 $$\begin{aligned} \omega _e = \omega _b + R_b \omega ^b_{eb}, \end{aligned}$$

where $\omega ^b_{eb}$ is the angular velocity of the end-effector relative to $\Sigma _b$ and is expressed in $\Sigma _b$.

Let $\Theta = [\theta _1, \theta _2]^T$ be the $(2 \times 1)$ vector of joint angles of the manipulator. The $(6 \times 1)$ vector of the generalized velocity of the end-effector with respect to $\Sigma _b$, $v^b_{eb} = [{\dot{p}}^{bT}_{eb},\omega ^{bT}_{eb}]^T$, can be expressed in terms of the joint velocities ${\dot{\Theta }}$ via the manipulator Jacobian $J^b_{eb}$^50^, such that

8 $$\begin{aligned} v^b_{eb} = J^b_{eb} {\dot{\Theta }}. \end{aligned}$$

From (6) and (7), the generalized end-effector velocity, $v_e = [{\dot{p}}^T_e, \omega ^T_e]^T$, can be expressed as

9 $$\begin{aligned} v_e = J_b v_b + J_{eb} {\dot{\Theta }}, \end{aligned}$$

where $$v_b = [{\dot{p}}^T_b,\omega ^T_b]^T$$, $$J_b= \begin{bmatrix} I_3 & -Skew(R_b p^b_{eb})\\ O_3 & I_3 \end{bmatrix},$$ $$J_{eb}= \begin{bmatrix} R_b & O_3\\ O_3 & R_b \end{bmatrix} J^b_{eb}$$,

where $I_m$ and $O_m$ denote $(m \times m)$ identity and $(m \times m)$ null matrices, respectively. If the attitude of the vehicle is expressed in terms of yaw-pitch-roll angles, then (9) will be

10 $$\begin{aligned} v_e = J_b Q_b \chi _b + J_{eb} {\dot{\Theta }}, \end{aligned}$$

with $$\chi _b= \begin{bmatrix} p_b \\ \Phi _b \end{bmatrix},$$ $$Q_b= \begin{bmatrix} I_3 & O_3\\ O_3 & T_b \end{bmatrix},$$ where $T_b$ describes the transformation matrix between the angular velocity $\omega _b$ and the time derivative of Euler angles ${\dot{\Phi }}_b$, and it is given as

11 $$\begin{aligned} T_b(\Phi _b)= \begin{bmatrix} 0 & -S(\psi ) & C(\psi ) C(\theta ) \\ 0 & C(\psi ) & S(\psi ) C(\theta ) \\ 1 & 0 & -S(\theta ) \\ \end{bmatrix}. \end{aligned}$$

Since the vehicle is an under-actuated system, i.e., only 4 independent control inputs are available for the 6-DOF system, the position and the yaw angle are usually the controlled variables. The pitch and roll angles are used as intermediate control inputs to control the horizontal position. Hence, it is worth rewriting the vector $\chi _b$ as follows $$\chi _b= \begin{bmatrix} \eta _b \\ \sigma _b \end{bmatrix},$$ $$\eta _b= \begin{bmatrix} p_b \\ \psi \end{bmatrix},$$ $$\sigma _b= \begin{bmatrix} \theta \\ \phi \end{bmatrix}.$$

Thus, the differential kinematics (10) will be

12 $$\begin{aligned} \begin{aligned} v_e&= J_{\eta } {\dot{\eta }}_b + J_{\sigma } {\dot{\sigma }}_b + J_{eb} {\dot{\Theta }}&=J_{\zeta } {\dot{\zeta }} + J_{\sigma } {\dot{\sigma }}_b, \end{aligned} \end{aligned}$$

where $\zeta = [\eta _b^T,\Theta ^T]^T$ is the vector of the controlled variables, $J_{\eta }$ is composed by the first 4 columns of $J_b Q_b$, $J_{\sigma }$ is composed by the last 2 columns of $J_b Q_b$, and $J_{\zeta } = [J_{\eta }, J_{eb}]$.

If the end-effector orientation is expressed via a triple of Euler angles, *ZYX*, $\Phi _e$, the differential kinematics (12) can be rewritten in terms of the vector ${\dot{\chi }}_e = [{\dot{p}}^T_e, {\dot{\Phi }}_e^T]^T$ as follows

13 $$\begin{aligned} \begin{aligned} {\dot{\chi }}_e&= Q_e^{-1}(\Phi _e) v_e&=Q_e^{-1}(\Phi _e) [J_{\zeta } {\dot{\zeta }} + J_{\sigma } {\dot{\sigma }}_b], \end{aligned} \end{aligned}$$

where $Q_e$ is the same as $Q_b$ but it is a function of $\Phi _e$ instead of $\Phi _b$.

### Dynamics

The equations of motion of the proposed robot have been derived in details in^21^. The dynamical model of the quadrotor-manipulator system can be reformulated in a matrix form as

14 $$\begin{aligned} M(q) \ddot{q} + C(q,{\dot{q}}) {\dot{q}} + G(q) + \tau _w + \tau _l=\tau , \qquad \tau = B u, \end{aligned}$$

where $q=[x, y, z, \psi , \theta , \phi , \theta _1, \theta _2]^T$ $\in R^{8}$ represents the vector of the generalized coordinates, *M* $\in R^{8 \times 8}$ denotes the symmetric and positive definite inertia matrix of the system, *C* $\in R^{8 \times 8}$ represents the Coriolis and centrifugal terms, *G* $\in R^{8}$ represents the gravity term, $\tau _w$ $\in R^{8}$ is vector of the external disturbances, $\tau _l$ $\in R^{8}$ is vector of the contact force effect, $\tau$ $\in R^{8}$ is the generalized input torques/forces, $u = [F_1, F_2, F_3, F_4, \tau _{m_1}, \tau _{m_2}]^T$ is vector of the actuator inputs, and $B= H N$ is the input matrix which is used to produced the body forces and moments from the actuator inputs. The control matrix, *N*, is given as

15 $$\begin{aligned} N= \begin{bmatrix} 0 & 0 & 0 & 0 & 0 & 0 \\ 0 & 0 & 0 & 0 & 0 & 0 \\ 1 & 1 & 1 & 1 & 0 & 0 \\ \gamma _1 & -\gamma _2 & \gamma _3 & -\gamma _4 & 0 & 0 \\ -d & 0 & d & 0 & 0 & 0 \\ 0 & -d & 0 & d & 0 & 0 \\ 0 & 0 & 0 & 0 & 1 & 0 \\ 0 & 0 & 0 & 0 & 0 & 1 \end{bmatrix}, \end{aligned}$$

where $\gamma _j=K_{m_j}/K_{f_j}$, and *H* $\in R^{8 \times 8}$ is matrix that transforms body input forces to be expressed in $\Sigma$ and is given by

16 $$\begin{aligned} H= \begin{bmatrix} R_b & O_3 & O_2 \\ O_3 & T_b^T R_b & O_2 \\ O_{2\times 3} & O_{2\times 3} & I_2 \end{bmatrix}. \end{aligned}$$

The environment dynamics, contact force, $\tau _l$, can be modeled as following:

17 $$\begin{aligned} \begin{aligned} \tau _l = J^T F_e,\qquad F_e= S_c \chi _e + D_c {\dot{\chi }}_e, \end{aligned} \end{aligned}$$

where $S_c = diag\{S_{c_1}, S_{c_2}, S_{c_3}, S_{c_4}, S_{c_5}, S_{c_6}\}$ and $D_c = diag\{D_{c_1}, D_{c_2}, D_{c_3}, D_{c_4}, D_{c_5}, D_{c_6}\}$ represent the environment stiffness and the environment damping, receptively.

The wind dynamics, $\tau _w$, can be modeled as following^51–53^:

The average wind velocity is determined by

18 $$\begin{aligned} V_{wz} = V_{w_{z_0}} \frac{z}{z_0}, \end{aligned}$$

where $V_{wz}$ is the wind velocity at altitude *z*, $V_{w_{z_0}}$ is the specified (measured) wind velocity at altitude $z_0$. To simulate wind disturbances, it is worth calculating the wind force, $F_{w}$, which influences the platform than the wind velocity. This force can be determined by

19 $$\begin{aligned} F_{w} = 0.61 * A_e V_{wz}^2, \end{aligned}$$

where 0.61 is used to convert wind velocity to pressure, and $A_e$ is the influence effective area which depends on the quadrotor structure and its orientation.

This force can be projected on the axes of frame $\Sigma$ as

20 $$\begin{aligned} \begin{aligned} F_{wx} = f_{wx_1} z^2 sin(\theta ) + f_{wx_2} z^2 cos(\theta ), \\ F_{wy} = f_{wy_1} z^2 sin(\phi ) + f_{wy_2} z^2 cos(\phi ), \end{aligned} \end{aligned}$$

where $f_{wx_1} = 0.61 * A_{e_1} (\frac{V_{w_{z_0}}}{z_0})^2 cos(\psi _w)$, $f_{wx_2} = 0.61 * A_{e_2} (\frac{V_{w_{z_0}}}{z_0})^2 cos(\psi _w)$, $f_{wy_1} = 0.61 * A_{e_1} (\frac{V_{w_{z_0}}}{z_0})^2 sin(\psi _w)$, $f_{wy_2} = 0.61 * A_{e_2} (\frac{V_{w_{z_0}}}{z_0})^2 sin(\psi _w)$, $\psi _w$ represents the angle of wind direction, and both $A_{e_1}$ and $A_{e_2}$ depend on the quadrotor design parameters.

## Controller design

### Control objectives

**Fig. 3:**
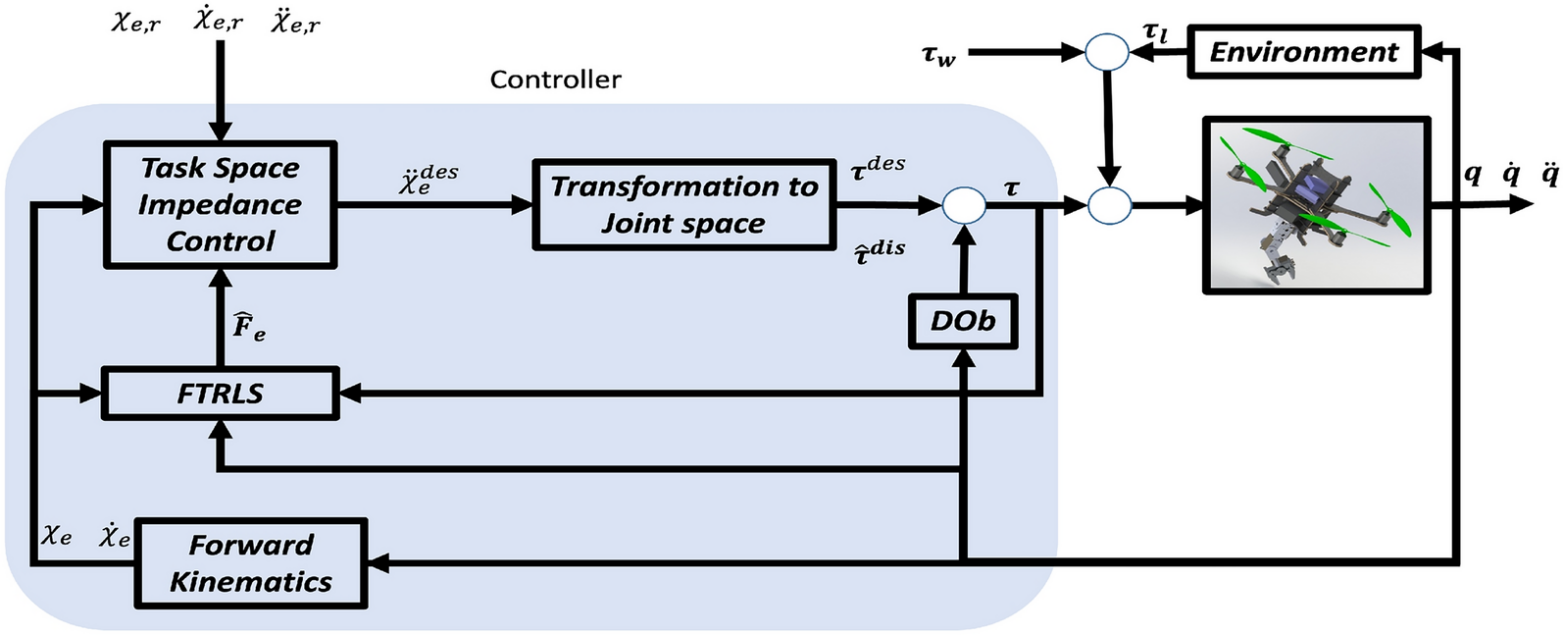
Block diagram of the proposed FTRLS-DOb control scheme for the quadrotor manipulation system.

Our goal is to design of estimation and control system to achieve the following objectives:

**1. Robust Stability:** The robotic system in Fig. 3 is robust and stable against the external disturbances, parameters uncertainties, and noises.

**2. Force Estimation:** The end-effector contact force has to be estimated with fast response and the estimation error tends to zero as the time tends to $\infty$.

**3. **6- **DOF Impedance Control: ** In the presence of the applied force/desired impedance at the end-effector, the end-effector tracking error tends to zero as time tends to $\infty$.

To this end, we propose a control scheme as shown in Fig. 3. In this control strategy, the system nonlinearities, external disturbances (wind), $\tau _{w}$, and contact force, $\tau _{l}$, are treated as disturbances, $\tau ^{dis}$, that will be estimated, ${\hat{\tau }}^{dis}$, and compensated by the DOb in the inner loop. The system can be now tackled as linear SISO plants. The output of DOb with system measurements of both joint and task spaces variables are used as the inputs to the FTRLS to obtain the end-effector contact force ${\hat{F}}_e$. The task space impedance control is used in the external loop of DOb and its output is transformed to the joint space through a transformation algorithm.

### Disturbance observer loop

**Fig. 4:**
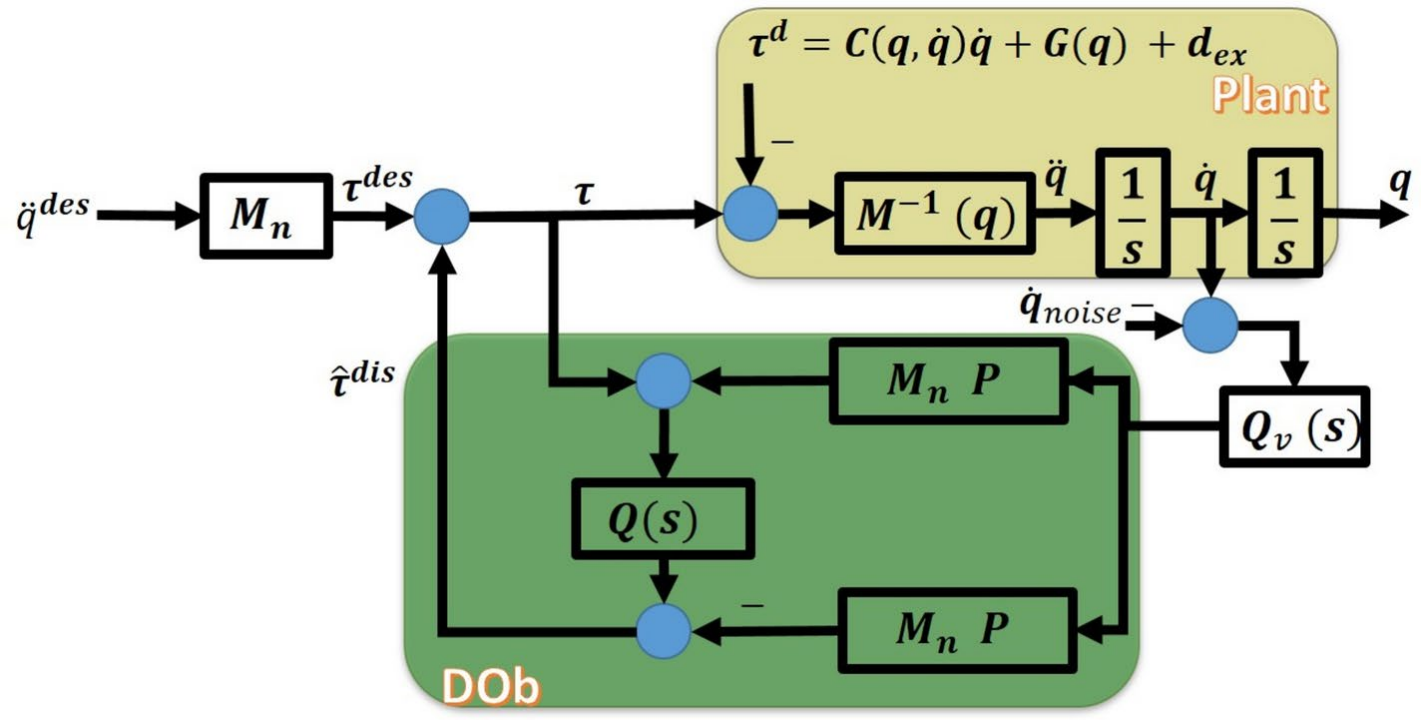
Block diagram of DOb internal loop.

A block diagram of the DOb inner loop is shown in Fig. 4. In this figure, $M_n$ $\in R^{8 \times 8}$ is the system nominal inertia matrix, $\tau$ and $\tau ^{des}$ are the robot and desired inputs, respectively, $P=diag([g_1,\ldots ,g_i,\ldots ,g_8])$ with $g_i$ is the bandwidth of the $i^{th}$ variable of *q*, $Q(s)=diag([\frac{g_1}{s+g_1} ,\ldots ,\frac{g_i}{s+g_i},\ldots ,\frac{g_8}{s+g_8}])$ $\in R^{8 \times 8}$ is the matrix of the low pass filter of DOb. The DOb requires velocity measurement. Practically, the velocity have to be fed through a low pass filter, $Q_v(s)=diag([\frac{g_{v_1}}{s+g_{v_1}},\ldots ,\frac{g_{v_i}}{s+g_{v_i}},\ldots ,\frac{g_{v_8}}{s+g_{v_8}}])$ $\in R^{8 \times 8}$, and with cut-off frequency of $P_v=diag([g_{v_1},\ldots ,g_{v_i},\ldots ,g_{v_8}])$. $\tau ^{dis}$ represents the system disturbances, and ${\hat{\tau }}^{dis}$ is the estimated disturbances.

If we apply the concept of disturbance observer to the proposed system, the independent coordinate control is possible without considering coupling effect of other coordinates. The coupling terms such as centripetal and Coriolis and gravity terms are considered as disturbance and compensated by feed forward the estimated disturbance torque.

The disturbance $\tau ^{dis}$ can be assumed as

21 $$\begin{aligned} \begin{aligned} \tau ^{dis} = (M(q) - M_n) \ddot{q} + \tau ^d,\\ \tau ^d=C(q,{\dot{q}}) {\dot{q}} + G(q) + d_{ex}. \end{aligned} \end{aligned}$$

Substituting from (21), then (14) can be rewritten as

22 $$\begin{aligned} M_n \ddot{q} + \tau ^{dis} = \tau . \end{aligned}$$

The control input, $\tau$, see Fig. 4, is given as

23 $$\begin{aligned} \begin{aligned} \tau = \frac{1}{(1-Q(s))}[M_n \ddot{q}^{des} - Q(s) M_n \ddot{q}] \quad = M_n \ddot{q}^{des} + M_n P e_v, \qquad e_v = {\dot{q}}^{des} - {\dot{q}}. \end{aligned} \end{aligned}$$

Applying this control law results in

24 $$\begin{aligned} \begin{aligned} M(q) {\dot{e}}_v + C(q,{\dot{q}}) e_v + K_v e_v = \delta , \qquad K_v = P M_n, \end{aligned} \end{aligned}$$

where

25 $$\begin{aligned} \begin{aligned} \delta = \Delta M(q) \ddot{q}^{des} + C(q,{\dot{q}}) {\dot{q}}^{des} + G(q) + d_{ex}, \qquad \Delta M(q) = M(q) - M_n. \end{aligned} \end{aligned}$$

Stability of this inner loop can be proved as following:

To simplify the analysis, let us ignore the effect of the velocity filter which will be considered later.

Let us use a Lyapunov function as

26 $$\begin{aligned} V=\frac{1}{2} e_v^T M(q) e_v. \end{aligned}$$

The time derivative of this function is

27 $$\begin{aligned} {\dot{V}}= e_v^T M(q) {\dot{e}}_v + \frac{1}{2} e_v^T {\dot{M}}(q) e_v. \end{aligned}$$

Substituting from (24), then (27) becomes

28 $$\begin{aligned} {\dot{V}}= e_v^T \delta - e_v^T K_v e_v + \frac{1}{2} e_v^T ({\dot{M}}(q) - 2 C(q,{\dot{q}}))e_v. \end{aligned}$$

To complete this proof, the properties of the dynamic equation of motion (14) will be utilized. These properties are^49,54^:

**property 1**

29 $$\begin{aligned} \lambda _{min} \Vert \nu \Vert ^2 \le \nu ^T M(q) \nu \le \lambda _{max} \Vert \nu \Vert ^2, \end{aligned}$$

**property 2**

30 $$\begin{aligned} \nu ^T({\dot{M}}(q) - 2 C(q,{\dot{q}}))\nu =0, \end{aligned}$$

where $\nu \in R^8$ represents a 8-dimensional vector, and $\lambda _{min}$ and $\lambda _{max}$ are positive real constants.

Substituting from (30), then (28) will be

31 $$\begin{aligned} {\dot{V}}= e_v^T \delta - e_v^T K_v e_v. \end{aligned}$$

From property (29), one can get

32 $$\begin{aligned} {\dot{V}} \le -\gamma V + \sqrt{\frac{2 V}{\lambda _{min}}} |\delta |, \quad \gamma = \frac{2K_v}{\lambda _{max}}. \end{aligned}$$

From the analysis presented in^55^, (32) can be reformulated as

33 $$\begin{aligned} \Vert e_v\Vert _p \le \frac{1}{\gamma } + \sqrt{\frac{2}{\lambda _{min}}} (\frac{2}{p\gamma })^{\frac{1}{p}} \sqrt{V(0,e_v(0))} \Vert \delta \Vert _p. \end{aligned}$$

Thus, the error dynamics is $L_p$ input/output stable with respect to the pair ($\delta$,$e_v$) for all $p \in [1,\infty ]$ with the assumption that the system states, *q* and ${\dot{q}}$, are bounded.

If one considers the effect of using the velocity filter, then the characteristic equation of the inner loop is

34 $$\begin{aligned} P_{c_i} = s^2 + g_{v_i} s + \alpha _i g_i g_{v_i}, \end{aligned}$$

where $$\alpha _i = \frac{M_{n_{ii}}}{M_{ii}}$$.

To improve the robustness, the damping coefficient of this equation, which is $0.5 \sqrt{\frac{g_i g_{v_i}}{\alpha g_i}}$, should larger than or equal 0.707 . Therefore, the following inequality

35 $$\begin{aligned} \alpha g_i \le \frac{g_{v_i}}{2}, \end{aligned}$$

should be hold. Recasting (35) with respect to $K_v$ gives to

36 $$\begin{aligned} \frac{K_{v_i} }{M_{ii}}\le \frac{g_{v_i}}{2}. \end{aligned}$$

Summarizing, (32) shows that the stability and robustness of the control system is enhanced by increasing $K_v$, i.e., by increasing $M_n$ and *P*, but without violating the robustness constraint given in (36). Equation (33) demonstrates that the error dynamics is stable and bounded. Equations (34-36) provide a method to determine the controller parameters, ensuring that the error converges to zero. Adhering to these conditions and performing fine-tuning will ensure that the convergence occurs and approaches zero closely.

If the DOb performs well, that is ${\hat{\tau }}^{dis}$ = $\tau ^{dis}$, the dynamics from the DOb loop input $\tau ^{des}$ to the output of the system is given as

37 $$\begin{aligned} M_n \ddot{q}=\tau ^{des}. \end{aligned}$$

Since $M_n$ is assumed to be a diagonal matrix, the system can be considered as a decoupled linear multi SISO systems as

38 $$\begin{aligned} M_{n_{ii}} \ddot{q}_i=\tau _i^{des}, \end{aligned}$$

or in the acceleration space as:

39 $$\begin{aligned} \ddot{q}_i=\ddot{q}_i^{des}. \end{aligned}$$

The next step is to design an Impedance tracking based controller in the outer loop for the system of (39).

### Fast tracking recursive least squares

In this part, we develop a technique which utilizes a Fast Tracking Recursive Least Squares (FTRLS) to estimate the contact force with the aid of the DOb linearization capabilities. The FTRLS algorithm is one of the fast online least squares-based identification methods used for the identification of environments with varying dynamic parameters^56,57^. FTRLS is one of the best methods for estimating these parameters in terms of accuracy, speed of estimation and computational cost. FTRLS strikes a balance between these three aspects, which is crucial for a quadrotor-based manipulation system due to its high dynamics. To apply FTRLS, the dynamic equations (14- 20) have to be parametrized (i.e., to be product of measurement data regressor and dynamic parameters) as follows:

The system dynamic part, $\tau _{int}= M(q) \ddot{q} + C(q,{\dot{q}}) {\dot{q}} + G(q)$, can be rewritten as the product of data regressor, $Y_i(q,{\dot{q}},\ddot{q})$, and platform parameters, $h_i$. The environment dynamics, $\tau _l$, can be reformulated as $Y_l(q,{\dot{q}},\ddot{q},\chi _e,{\dot{\chi }}_e)*h_l$, where, $Y_l=J^T Y_e$, $Y_e$ is a function of the end effector states, ($\chi _e$,${\dot{\chi }}_e$), and $h_l$ is the environment parameters $S_c$ and $D_c$. Finally, the wind effect is formulated as $Y_w(z,\theta ,\phi ) * h_w$, where $h_w$ is the wind parameters. Thus, the total dynamics can be reformulated as

40 $$\begin{aligned} \begin{aligned} \tau = Y * h, \qquad Y=[Y_i, Y_l, Y_w], \qquad h=[h_i, h_l, h_w]^T, \end{aligned} \end{aligned}$$

where *Y* $\in R^{8 \times 40}$ and *h* $\in R^{40}$ are the data regressor and parameters vector of (14), respectively.

The parameter estimation error is

41 $$\begin{aligned} {\tilde{h}}(t) = h - {\hat{h}}(t), \end{aligned}$$

while the estimation error is

42 $$\begin{aligned} {\tilde{\tau }}(t) = \tau (t) - Y(t) {\hat{h}}(t) = Y(t) {\tilde{h}}(t). \end{aligned}$$

By minimizing a cost function with respect to the parameter estimation error, one can find the time derivative of the estimated parameters vector, ${\hat{h}}$, as following

43 $$\begin{aligned} \frac{d}{dt}{\hat{h}}(t)= R(t) Y^{T}(t) {\tilde{\tau }}(t), \end{aligned}$$

where *R*(*t*) is the parameters’ covariance matrix, and it can be calculated from

44 $$\begin{aligned} \frac{d}{dt}R^{-1}(t) = -\eta _h(t) R^{-1}(t) + Y^{T}(t) Y(t), \end{aligned}$$

where $\eta _h$ is the forgetting factor, and it is given as

45 $$\begin{aligned} \eta _h(t) = \eta _h^{min} + (1-\eta _h^{min}) 2^{(-NINT(\gamma _g \Vert {\tilde{\tau }}(t)\Vert ^2))}, \end{aligned}$$

where $\eta _h^{min}$ is a constant representing the minimum forgetting factor, *NINT*(.) is the round-off operator, and $\gamma _g$ is a design constant. This adaptive formulation of the forgetting factor enables the RLS to track the non-stationary parameters to be estimated.

The convergence/stability (${\tilde{h}}(t) \longrightarrow 0$) proof of this algorithm can be implemented as following:

Let us assume the Lyapunov function as

46 $$\begin{aligned} V(t) = {\tilde{h}}^T(t) R^{-1}(t) {\tilde{h}}(t). \end{aligned}$$

If $R^{-1}(t)$ is chosen to be positive definite, then *V*(*t*) will be positive definite. To prove the positive definiteness of $R^{-1}(t)$, let us use the solution of the differential equation (44) which is

47 $$\begin{aligned} R^{-1}(t) = \Phi _h(t,t_0) R^{-1}(t_0) \Phi _h^T(t,t_0) + \int _{t_0}^{t} \Phi _h(t,\varrho ) Y^T(\varrho ) Y(\varrho ) \Phi _h^T(t,\varrho ) d\varrho , \end{aligned}$$

where $\Phi _h^T(t,t_0)$ is the state transition matrix of a system described by ${\dot{\upsilon }}(t) = - \frac{1}{2}\eta _h \upsilon (t)$. Thus, by choosing $R^{-1}(t_0)> 0$, then the first term in (47) will be positive definite. The second term is also positive definite. As a result, the proposed covariance matrix update formula is positive definite, and thus, the chosen Lyapunov function (46) is positive definite.

The time derivative of Lyapunov function is

48 $$\begin{aligned} {\dot{V}}(t) = 2 {\tilde{h}}^T R^{-1} \dot{{\tilde{h}}} + {\tilde{h}}^T \dot{R^{-1}} {\tilde{h}}. \end{aligned}$$

However, by differentiating both sides of (41) with respect to time, one can find that $\dot{{\tilde{h}}} = - \dot{{\hat{h}}}$, by substituting from the proposed formula of $\dot{{\hat{h}}}$ (43) and (42), then

49 $$\begin{aligned} \dot{{\tilde{h}}} = - R Y^T Y {\tilde{h}}. \end{aligned}$$

Substituting from (49) in (48), then ${\dot{V}}(t)$ will be

50 $$\begin{aligned} {\dot{V}}(t) = -{\tilde{h}}^T [2 Y^T Y - \dot{R^{-1}}] {\tilde{h}}. \end{aligned}$$

Substituting from the proposed formula (44) for $\dot{R^{-1}}$ into (50), then

51 $$\begin{aligned} {\dot{V}}(t) = -{\tilde{h}}^T [Y^T Y +\eta _h(t) R^{-1}(t)] {\tilde{h}}. \end{aligned}$$

Thus, the time derivative of *V*(*t*) is negative definite which ensures the asymptotic stability of the estimation error (${\tilde{h}}(t) \longrightarrow 0$ as $t \longrightarrow \infty$)

Finally, for both teleoperation impedance control purposes, the user can calculate the estimated environment impedance, contact force, from

52 $$\begin{aligned} \begin{aligned} {\hat{\tau }}_l = Y_l {\hat{h}}_l,\qquad {\hat{F}}_e = Y_e {\hat{h}}_l. \end{aligned} \end{aligned}$$

Therefore, unlike the current developed schemes, with this technique, one can isolate and estimate the end-effector contact force apart from the whole estimated forces in the systems.

### Impedance control

The objective of the impedance control is to regulate the end-effector interaction force, which may vary due to the uncertainty in the location of the interaction point and/or the structural properties of the environment, besides achieving task space trajectory tracking. The linear impedance control is designed in the task space. This is based on the linearization effect of the designed DOb in the joint space. The desired acceleration in the task space, $\ddot{\chi }^{des}_e$, can be calculated from

53 $$\begin{aligned} \ddot{\chi }^{des}_e = \ddot{\chi }_{e,r} + S_{c,d} (\chi _{e,r} - \chi _{e}) + D_{c,d} ({\dot{\chi }}_{e,r} - {\dot{\chi }}_{e}) - {\hat{F}}_e, \end{aligned}$$

where $S_{c,d}$ and $D_{c,d}$ are the desired values of $S_{c}$ and $D_{c}$ respectively, which determine the desired impedance that the end-effector will apply to the environment. Let us define the quadrotor/joint space tracking error as

54 $$\begin{aligned} e = q_r - q, \qquad {\dot{e}} = {\dot{q}}_r - {\dot{q}}, \qquad \ddot{e} = \ddot{q}_r - \ddot{q}, \end{aligned}$$

while the task space tracking error can be defined as

55 $$\begin{aligned} e_e = \chi _{e,r} - \chi _{e}, \qquad {\dot{e}}_e = {\dot{\chi }}_{e,r} - {\dot{\chi }}_{e}, \qquad \ddot{e}_e = \ddot{\chi }_{e,r} - \ddot{\chi }_{e}, \end{aligned}$$

where $\chi _{e,r}$, ${\dot{\chi }}_{e,r}$, and $\ddot{\chi }_{e,r}$ are the reference trajectories for the position, velocity, acceleration in the task space, respectively which are chosen to be bounded and continuous. $q_r$, ${\dot{q}}_{r}$, and $\ddot{q}_{r}$ are the reference trajectories for the position, velocity, acceleration in the quadrotor/joint space, respectively. Transformation from the task space to quadrotor/joint space will be implemented via the inverse of system Jacobian. The relation between the inner loop and the outer loop errors can be obtained as follows. The DOb loop error can be expressed in the task space, $e_{v,e}$, via the Jacobian by

56 $$\begin{aligned} e_{v,e} = J e_v, \end{aligned}$$

where $e_{v,e} = {\dot{\chi }}_e^{des} - {\dot{\chi }}_e$. From the previous analysis, it is proved that $e_v$ is bounded as in (33). If we define ${\dot{e}}_{v,e} = \ddot{\chi }_e^{des} - \ddot{\chi }_e$, then by substituting from (53), one can get

57 $$\begin{aligned} {\dot{e}}_{v,e} = \ddot{e}_e + S_{c,d} e_e + D_{c,d} {\dot{e}}_e - {\hat{F}}_e. \end{aligned}$$

Equation (57) can be reformulated in a state space form as

58 $$\begin{aligned} {\dot{X}}_e = A_e X_e + B_e U_e, \end{aligned}$$

where $$X_e = \begin{bmatrix} e_e\\ {\dot{e}}_e\\ \end{bmatrix}$$, $$A_e = \begin{bmatrix} O_6 & I_6\\ - S_{c,d} & -D_{c,d} \end{bmatrix}$$, $$B_e = \begin{bmatrix} O_6\\ I_6\\ \end{bmatrix}$$, and $U_e = {\dot{e}}_{v,e} + {\hat{F}}_e$. By inspecting the matrix $A_e$ and based on the boundedness of both ${\dot{e}}_{v,e}$ and ${\hat{F}}_e$, one can find that the state, $X_e = [ e_e^T, {\dot{e}}_e^T]^T$, is bounded and exponentially tends to zero as time tends to infinity as soon as the matrices, $S_{c,d}$ and $D_{c,d}$, are positive definite. As a result, since the Jacobian inverse exists (no singularities), the system errors, *e* and ${\dot{e}}$, are also bounded and exponentially tends to zero as time tends to infinity.

**Fig. 5:**
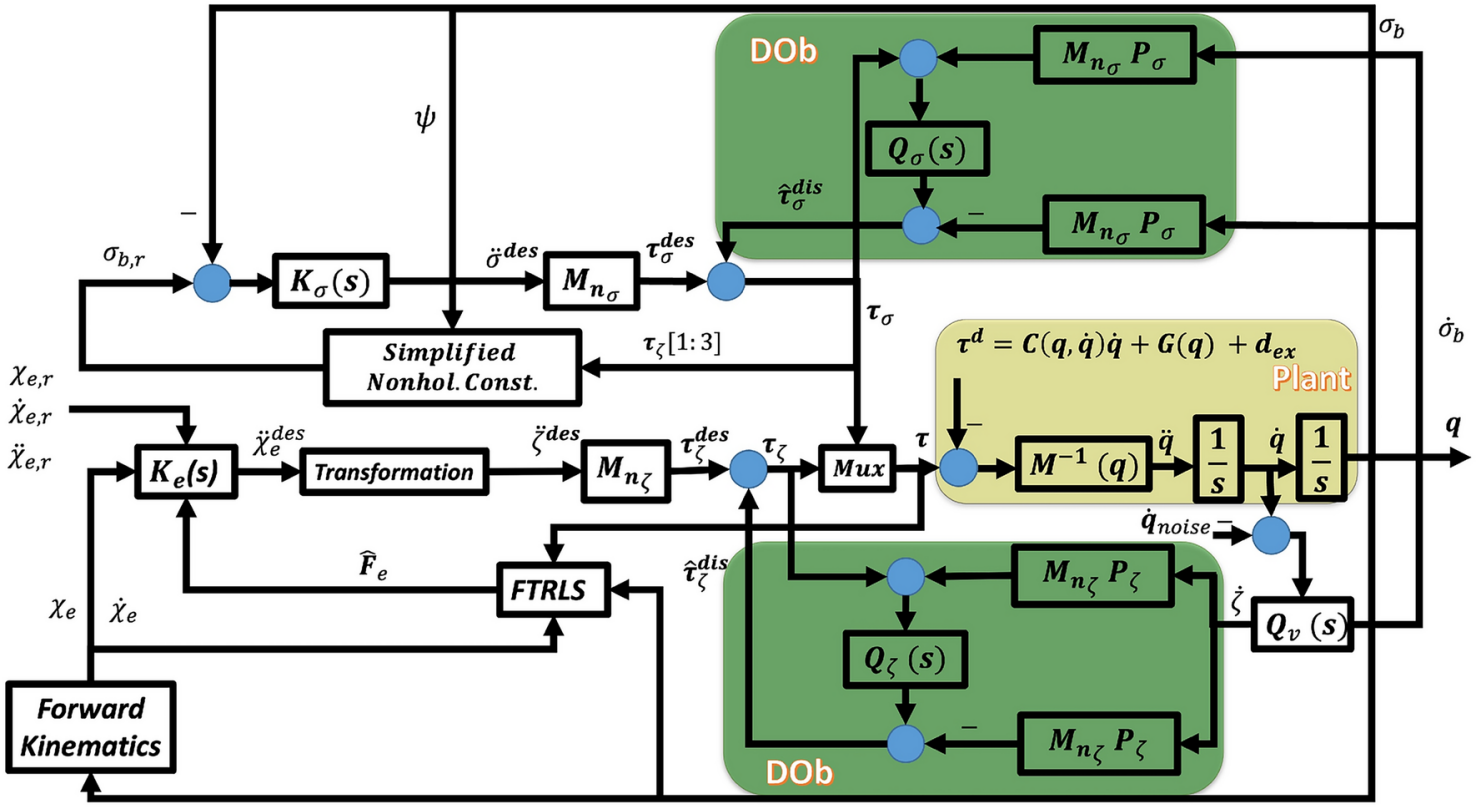
Block diagram of the detailed FTRLS-DOb control scheme for the quadrotor manipulation system.

A complete and detailed block diagram of the proposed control scheme is illustrated in Fig. 5. Quadrotor position and yaw rotation are the controlled variables, while pitch and roll angles are used as intermediate control inputs to achieve the desired *x* and *y*. Therefore, the proposed scheme has two DOb-based controllers include one for $\zeta =[x, y, z, \psi , \theta _1, \theta _2]^T$ (with $M_{n_\zeta }$, $P_{\zeta }$, $Q_{\zeta }$) and the other for $\sigma _b = [\theta , \phi ]^T$ (with $M_{n_\sigma }$, $P_{\sigma }$, $Q_{\sigma }$). The desired 6-DOF trajectories for the end-effector’s ($\chi _{e,r}$), their actual values calculated by the forward kinematics, and the estimated end-effector force, are applied to the impedance control algorithm, $K_e$ that is given in (53). Then, a transformation from task space to joint space is done by using (59) to get $\ddot{\zeta }^{des}$. The desired acceleration in the joint space, $\ddot{\zeta }^{des}$, can be calculated by differentiating (13) with respect to time as

59 $$\begin{aligned} \ddot{\zeta }^{des} = J_{\zeta }^{-1} (Q_e \ddot{\chi }^{des}_e + {\dot{Q}}_e {\dot{\chi }}_{e,r} - {\dot{J}}_{\zeta } {\dot{\zeta }} - J_{\sigma } \ddot{\sigma }_b - {\dot{J}}_{\sigma } {\dot{\sigma }}_b), \end{aligned}$$

The desired acceleration in quadrotor/joint space, $\ddot{\zeta }^{des}$, is then applied to the DOb of the independent coordinates, $\zeta$, to produce $\tau _{\zeta }$. The desired values for the intermediate DOb controller, $\sigma _{b,r}$, are obtained from the output of position controller, $\tau _{\zeta }$, through the following simplified nonholonomic constraints relation

60 $$\begin{aligned} \sigma _{b,r} = \frac{1}{\tau _{\zeta }(3)} \begin{bmatrix} C(\psi ) & S(\psi ) \\ S(\psi ) & -C(\psi ) \end{bmatrix} \begin{bmatrix} \tau _{\zeta }(1) \\ \tau _{\zeta }(2) \end{bmatrix}. \end{aligned}$$

The external controller of second DOb controller, $\tau _{\sigma }$, is used as a PD controller with velocity feedback, $K_{\sigma }$, as following:

61 $$\begin{aligned} \ddot{\sigma }^{des} = K_{p_{\sigma }} (\sigma _{b,r} - \sigma _{b}) - K_{d_{\sigma }} {\dot{\sigma }}_{b}. \end{aligned}$$

After that, $\ddot{\sigma }^{des}$ is applied to the second DOb to generate $\tau _{\sigma }$.

It is konwn that the response of the $\sigma$ controller must be much faster than that of the position controller. This can be achieved by the tuning parameters of both DOb and PD of $\sigma$-controller.

The output of two controllers are converted to the required forces/torques applied to quadrotor/manipulator by

62 $$\begin{aligned} u= B_6^{-1} \begin{bmatrix} \tau _{\zeta }(3,4)\\ \tau _{\sigma }\\ \tau _{\zeta }(5,6) \end{bmatrix}, \end{aligned}$$

where $B_6$ $\in R^{6 \times 6}$ is part of *B* matrix and it is given by $B_6 = B(3:8,1:6)$.

## Simulation results

In this section, the presented aerial manipulation robot model with the proposed control technique is implemented in MATLAB/SIMULINK.

### Simulation environment

**Fig. 6:**
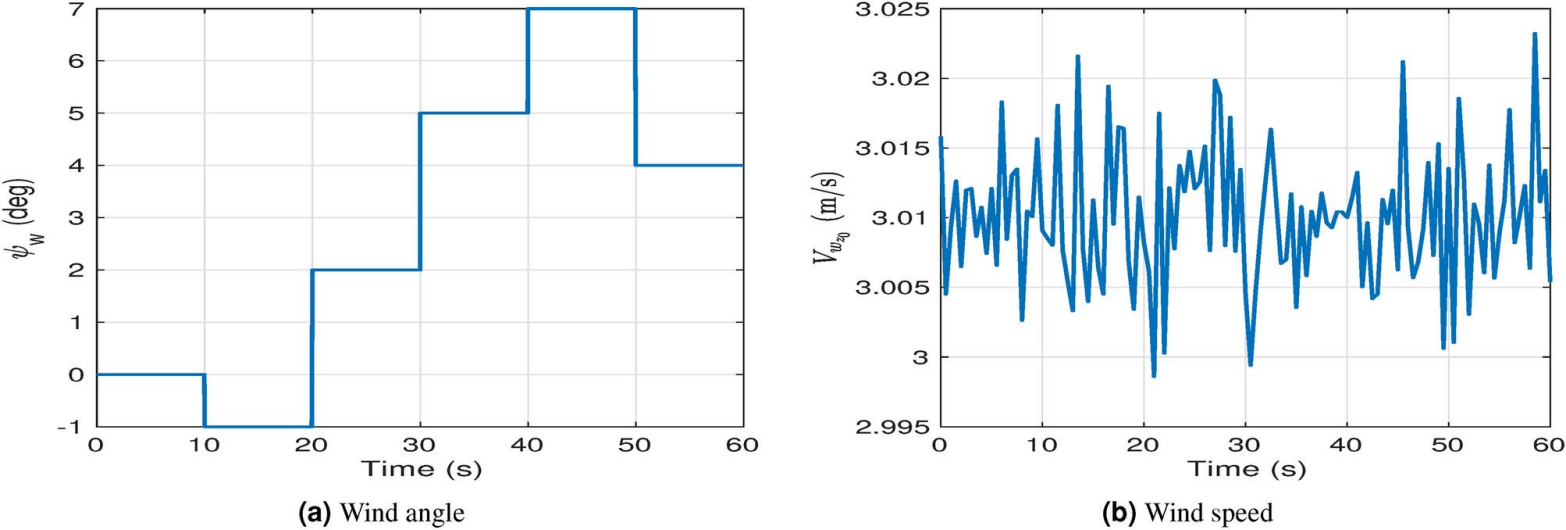
Profile of the wind: (**a**) angle, and (**b**) speed.

For a more realistic simulation studies, the following setup have been made:

- Linear and angular position and orientation of the quadrotor are available at rate of 1 KHz. In^58^, a scheme is proposed to measure and estimate the vehicle (Asctec Pelican Quadrotor) states based on IMU and Onboard camera in both indoors and outdoors.
- The joints angles are measured at rate of 1 KHz and angular velocities are estimated by a low pass filter.
- The measured signals are affected by a normally distributed measurement noise with mean of $10^{-3}$ and standard deviation of $5 \times 10^{-3}$.
- 1 KHz Control loop.
- To test the robustness against model uncertainties, a step disturbance is applied at 15 s to both the inertia matrix, *M*(*q*), and the control matrix, *N*, (Actuators’ losses) with $10 \%$ error.
- A wind disturbance which is a time-varying is added. The wind angle profile, $\psi _w$, is simulated as shown in Figure 6.a. To replicate a real-world scenario, the value of the wind velocity $V_{w_{z_0}}$ is composed of two components: a constant part and a random variable part to simulate gust effects (sudden and random changes in wind speed), as depicted in Figure 6.b.

**Table 1:** System Parameters.

Par.	Value	Unit	Par.	Value	Unit
*m*	1	*kg*	$L_2$	$85\times 10^{-3}$	*m*
*d*	$223 \times 10^{-3}$	*m*	$m_0$	$30\times 10^{-3}$	*kg*
$I_x$	$13.2 \times 10^{-3}$	$N.m.s^2$	$m_1$	$55\times 10^{-3}$	*kg*
$I_y$	$12.5 \times 10^{-3}$	$N.m.s^2$	$m_2$	$112\times 10^{-3}$	*kg*
$I_z$	$23.5 \times 10^{-3}$	$N.m.s^2$	$I_r$	$33.2 \times 10^{-6}$	$N.m .s^2$
$L_0$	$30\times 10^{-3}$	*m*	$L_1$	$70\times 10^{-3}$	*m*
$K_{F_1}$	$1.6\times 10^{-5}$	$kg.m.rad^{-2}$	$K_{F_2}$	$1.2\times 10^{-5}$	$kg.m.rad^{-2}$
$K_{F_3}$	$1.7\times 10^{-5}$	$kg.m.rad^{-2}$	$K_{F_4}$	$1.5\times 10^{-5}$	$kg.m.rad^{-2}$
$K_{M_1}$	$3.9\times 10^{-7}$	$kg.m^{2}.rad^{-2}$	$K_{M_2}$	$2.8\times 10^{-7}$	$kg.m^{2}.rad^{-2}$
$K_{M_3}$	$4.4\times 10^{-7}$	$kg.m^{2}.rad^{-2}$	$K_{M_4}$	$3.1\times 10^{-7}$	$kg.m^{2}.rad^{-2}$

The realistic system parameters are introduced in Table 1. The desired trajectories of the end-effector are generated to follow a circular helix, while its orientation follows quintic polynomial trajectories^49^. Parameters of the proposed algorithm are presented in Table 2. The controller is tested to achieve task space trajectory tracking under the effect of the contact force, wind disturbances, and measurement noise.

The aim of considered case study is to perform tracking of the desired 6-DOF end-effector trajectories (i.e., circular helix for the position and Quintic trajectories for the orientation) while an external force of 1.6 N acts along the z-axis of the end-effector, $z_e$, for $z_e \le 10$ cm. This scenario simulates, for instance, the case of the manipulator in contact with a soft environment. Thereby, the manipulator end-effector is chosen to be rigid. Hence the stiffness matrix, $S_{c,d}$, has been tuned to $S_{c,d}$ = $diag\{20, 20, 30, 50, 100, 500\}$, while the damping matrix value is $D_{c,d}$ = $diag\{15, 15, 25, 100, 100, 100\}$.

**Table 2:** Controller parameters.

*Parameter*	*Value*	*Par*.	*Val*.
$M_{n_\zeta }$	$diag\{0.02, 0.02, 2, 0.05, 0.01, 0.01\}$	$A_{e_1}$	0.16
$S_{c,d}$	$diag\{20, 20, 30, 50, 100, 500\}$	$D_{c}$	$0.01 I_6$
$D_{c,d}$	$diag\{15, 15, 25, 100, 100, 100\}$	$g_{v_i}$	100
$M_{n_\sigma }$	$diag\{0.05, 0.05\}$	$A_{e_2}$	0.032
$\eta ^{min}_{h}$	0.8	$\gamma _{g}$	5
$S_{c}$	$0.1 I_6$	$z_0$	1
$K_{p_\sigma }$ / $K_{d_\sigma }$	$20 I_2$	$V_{w_{z_0}}$	3

**Fig. 7:**
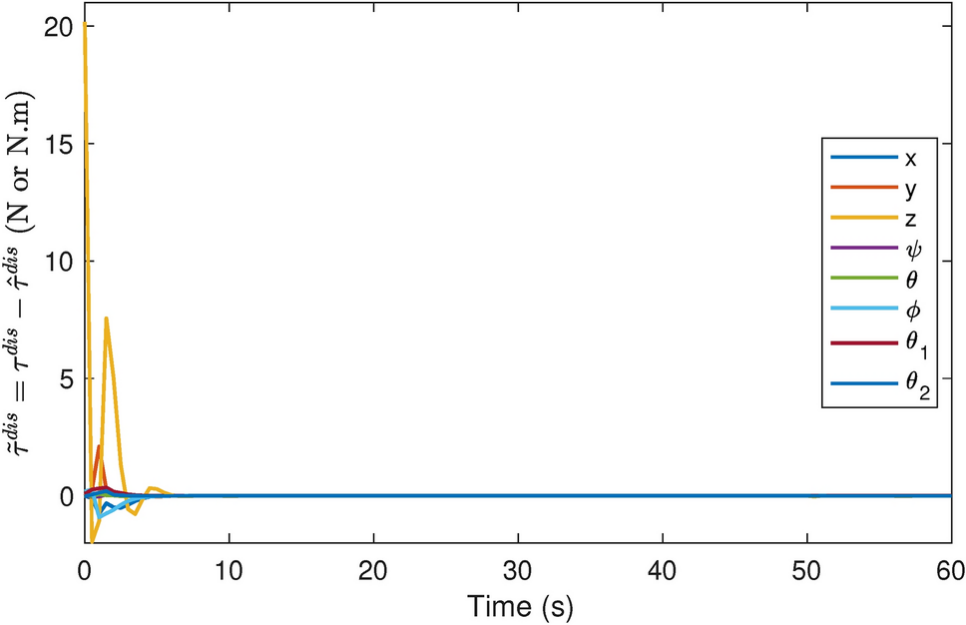
Error of disturbances estimation.

Figure 7 shows the error in disturbances estimation. The figure clearly indicates that the estimation error is highest at the beginning of the operation. This initial peak is due to the time needed for the DOb to accurately estimate the system dynamics, disturbances, and external forces. After this initial phase, the disturbance estimation error gradually vanishes. The figure demonstrates the effective estimation of disturbances, confirming that the proposed control scheme successfully estimates the disturbances.

### Estimation of the end-effector contact force

**Fig. 8:**
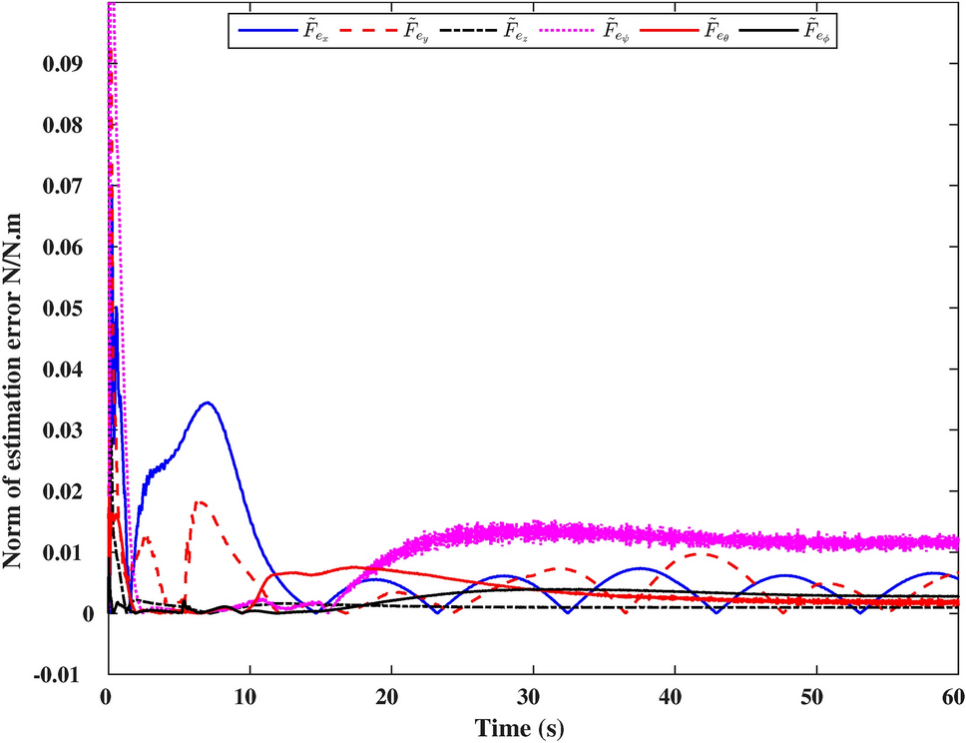
Error norm of estimation of the environment dynamics/contact force.

Figure 8 illustrates the response of the proposed algorithm in estimating the environmental effect/end-effector contact force. It is evident from the figure that the norm of the end-effector generalized force reaches a maximum value of 0.1 N/N.m at the start of the operation. This peak occurs due to the time required by the DOb to accurately estimate the system dynamics and external forces. This initial period lasts approximately 3 seconds, after which the error norm gradually decreases. The norm of the estimation error in both the *x* and *y* directions reaches a maximum value of 0.03 N, exhibiting a sinusoidal pattern due to the sinusoidal motion in these axes. In contrast, the norm value in the *z* direction peaks at 0.005 N. In both the $\phi$ and $\theta$ directions, the norms reach a value of 0.007 N.m. The maximum norm value in the $\psi$ direction is approximately 0.0015 N.m. These results highlight the effective estimation performance of the end-effector generalized forces. Therefore, it can be concluded that the second control objective has been successfully achieved.

### Impedance control

**Fig. 9:**
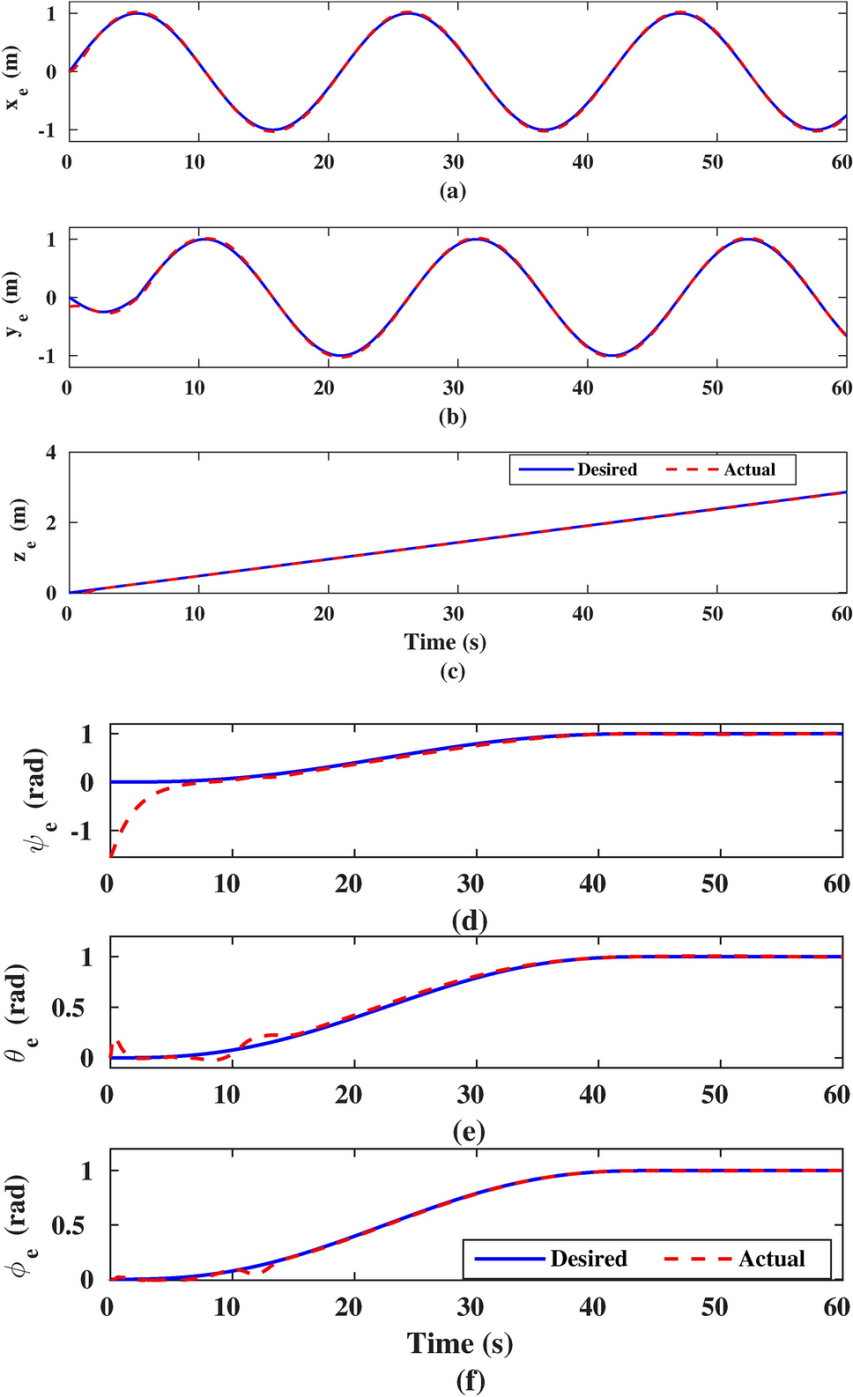
The actual response of the end-effector position and orientation: (**a**) $x_{e}$, (**b**) $y_{e}$, (**c**) $z_{e}$, (**d**) $\psi _{e}$, (**e**) $\theta _{e}$, and (**f**) $\phi _{e}$.

Figure 9 depicts the system’s response in the task space, where the actual end-effector position and orientation are determined through forward kinematics. The figure demonstrates that the controller effectively tracks the desired end-effector trajectories, with the tracking error approaching zero over time. Additionally, it highlights the proposed technique’s ability to maintain trajectory tracking despite parameter uncertainties introduced at instant 15 seconds. As observed, there is no impact on tracking in the *x*, *y*, *z*, and $\psi$ directions. However, in the $\theta$ and $\phi$ directions, the effects of uncertainty are noticeable, but the controller quickly recovers the tracking.

**Fig. 10:**
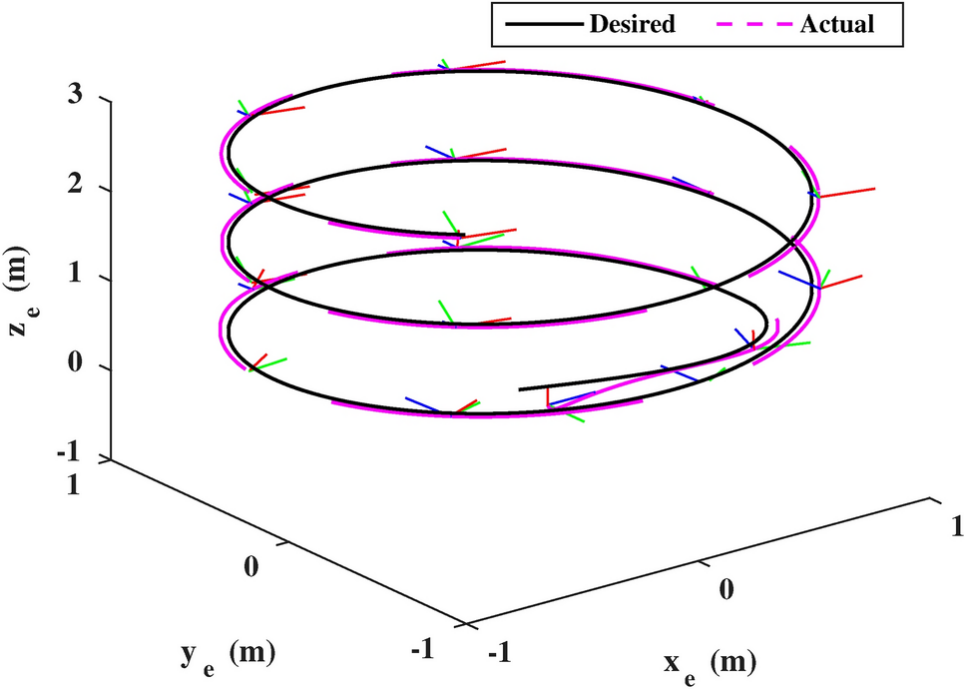
3D trajectory of the end-effector pose (The marker represents the end-effector orientation; Green, Blue, and Red for x-,y, and z-axis, respectively).

Figure 10 illustrates the end-effector’s motion in the 3D dimensions, with markers indicating orientation. The results demonstrate that the proposed impedance motion control scheme effectively tracks the desired end-effector trajectories and achieves the desired compliance/impedance effect on the environment, even in the presence of external disturbances and noise. Consequently, it can be asserted that the three control objectives have been successfully achieved.

## Conclusion

The problem of the contact force estimation and impedance control of an aerial manipulation robot is presented with a new solution. A brief presented of the system modeling is given. DOb-based system linearization is implemented in the quadrotor/joint space. A DOb is used in the inner loop is to achieve robust linear input/output behavior of the system by compensating disturbances, measurement noise, and uncertainties. Contact force/environment impedance is estimated based on FTRLS and DOb which appear efficient estimation results and stability guarantee. Then, a linear impedance control is designed and implemented in the task space. The inverse kinematics problem is solved by utilization of the system Jacobian. The controller is tested to achieve trajectory tracking under the effect of external wind disturbances, parameters uncertainty, and measurement noise. Numerical results enlighten the efficiency of the proposed control scheme.

## Data Availability

The datasets used and/or analyzed during the current study available from the corresponding author on reasonable request.

## Declarations

### Accession codes

Correspondence and requests for materials should be addressed to Alaa Khalifa.
